# Assessing Crystallisation Kinetics of Zr Metal–Organic Frameworks through Turbidity Measurements to Inform Rapid Microwave‐Assisted Synthesis

**DOI:** 10.1002/chem.202000993

**Published:** 2020-05-11

**Authors:** Sarah L. Griffin, Maria L. Briuglia, Joop H. ter Horst, Ross S. Forgan

**Affiliations:** ^1^ WestCHEM School of Chemistry University of Glasgow Glasgow UK; ^2^ EPSRC Centre for Innovative Manufacturing in, Continuous Manufacturing and Crystallisation (CMAC) Strathclyde Institute of Pharmacy and Biomedical Sciences, Technology and Innovation Centre University of Strathclyde 99 George Street Glasgow UK

**Keywords:** coordination modulation, metal–organic frameworks, microwave synthesis, nucleation, turbidity measurements

## Abstract

Controlling the crystallisation of metal‐organic frameworks (MOFs), network solids of metal ions or clusters connected by organic ligands, is often hindered by the significant number of synthetic variables inherent to their synthesis. Coordination modulation, the addition of monotopic competing ligands to solvothermal syntheses, can allow tuning of physical properties (particle size, porosity, surface chemistry), enhance crystallinity, and select desired phases, by modifying the kinetics of self‐assembly, but its mechanism(s) are poorly understood. Herein, turbidity measurements were used to assess the effects of modulation on the solvothermal synthesis of the prototypical Zr terephthalate MOF UiO‐66 and the knowledge gained was applied to its rapid microwave synthesis. The studied experimental parameters—temperature, reagent concentration, reagent aging, metal precursor, water content, and modulator addition—all influence the time taken for onset of nucleation, and subsequently allow microwave synthesis of UiO‐66 in as little as one minute. The simple, low cost turbidity measurements align closely with previously reported in situ synchrotron X‐ray diffraction studies, proving their simplicity and utility for probing the nucleation of complex materials while offering significant insights to the synthetic chemist.

## Graphical abstract

**Simple turbidity measurements** elucidate the effects of additives and modulators on the rates of nucleation and crystallisation of the archetypal Zr MOF UiO‐66, correlating with previous in situ synchrotron diffraction measurements, and informing rapid microwave syntheses while explaining experimental observations of physical properties.

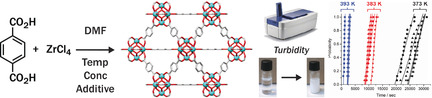

## Introduction

Despite the growth in interest and vast number of metal‐organic frameworks (MOFs)—coordination polymers wherein organic ligands connect metal ions or clusters into network solids—synthesised over recent years, there has been relatively little investigation into the crystallisation process of these materials. As a result, understanding of the reaction and crystallisation mechanisms remains limited, with MOF synthesis often proceeding more through trial and error than by design.^1^, ^2^ Understanding how these materials assemble would allow a rational approach towards MOF design and synthesis, increasing the ability to generate highly specialised materials of desired size, topology and functionality.^3^, ^4^, ^5^

**Figure 1:**
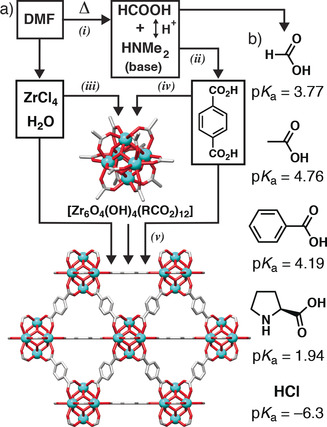
a) Schematic of variables to consider in the self‐assembly of UiO‐66: *(i)* the thermal decomposition of DMF to release base and formic acid, a potential coordination modulator, with associated proton balance (temperature); *(ii)* the deprotonation of the 1,4‐benzenedicarboxylate (BDC) linker (pH); *(iii)* the formation of the hexanuclear [Zr_6_O_4_(OH)_4_(RCO_2_)_12_] secondary building unit (Zr source, water content); *(iv)* the level of incorporation of the BDC linker in these SBUs and competition from additional ligands (modulators); *(v)* final substitution of transient ligands/modulators to allow coalescence into the UiO‐66 structure, redrawn from CCDC deposition RUBTAK,^16^ and its subsequent nucleation (reagent concentration). b) Typical coordination modulators used in the self‐assembly of Zr MOFs.

Crystallisation is known to heavily depend on various different reaction parameters, such as temperature, pH and time,^6^, ^7^ whilst addition of crystallisation promoters has been shown to increase crystallinity and allow particle size control.^8^, ^9^, ^10^, ^11^ An alternative is the use of coordination modulation—the addition of monotopic ligands, known as modulators, into synthetic mixtures to compete with the linkers for metal coordination, allowing fine control over a number of physical properties such as size, morphology, defectivity and porosity in MOFs linked by high valent metals—a technique now ubiquitous in the self‐assembly of Zr MOFs.^12^, ^13^, ^14^, ^15^ Knowledge on precisely how, why and to what extent these parameters affect crystallisation is limited however. As summarised in a recent review by Van Vleet et al., several in situ and ex situ techniques have been used to probe the nucleation and growth of MOFs.^2^ An in situ total X‐ray scattering study carried out by Xu et al. examined the formation of the hexanuclear Zr_6_ secondary building units (SBU) during the solvothermal synthesis of the archetypal zirconium terephthalate MOF UiO‐66^16^ (Figure 1 a), [Zr_6_O_4_(OH)_4_(BDC)_6_] (BDC=1,4‐benzenedicarboxylate), suggesting self‐assembly occurs by the formation of SBUs in a metal salt precursor solution, followed by assembly of multinuclear clusters upon the addition of the organic linkers, and finally coalescence of these clusters to form an ordered crystalline framework.^17^ An in situ synchrotron energy dispersive X‐ray diffraction (EDXRD) study into the formation of the isoreticular Zr‐fum analogue, [Zr_6_O_4_(OH)_4_(fumarate)_6_], in water showed that the addition of increasing equivalents of formic acid modulator to the aqueous synthesis results in a decrease in both the nucleation rate and crystal growth rate, supporting the concept of linker/modulator competition decelerating the reaction rate (typical modulators are shown in Figure 1 b).^18^

More recently, in situ EDXRD has been used to examine the impact of the organic linker and modulators on the crystallisation kinetics of UiO‐66(Zr/Hf)‐type MOFs, with results indicating the crystallisation process to be highly complex, as linker length and solubility, and modulator p*K*_a_, all influence crystallisation.^19^ Water has been shown to speed up the nucleation of UiO‐66, presumably by providing a source of the bridging O^2−^ and OH^−^ ligands found in the Zr_6_ SBU, while concentrated HCl (37 % *w/w* aqueous) slows nucleation compared to pure water, presumably by disfavouring linker deprotonation, but nucleation is still faster than unmodulated syntheses due to the incipient water.^20^ Addition of aqueous HCl to solvothermal syntheses has also been found to improve crystallinity of the resulting Zr MOFs.^21^

The majority of the current studies into crystallisation processes of MOFs are carried out under solvothermal conditions. There are a variety of synthesis methods for MOFs, however, with microwave assisted heating becoming a commonly used alternative given its ability to vastly reduce synthesis times (and consequently lower energy consumption), allow for control of particle properties, such as size and morphology, and produce otherwise unobtainable materials.^22^, ^23^, ^24^, ^25^ The use of microwave heating does, however, introduce a new area of uncertainty in the crystallisation of MOFs. As rapid microwave heating offers a different route for energy to be introduced into the system—the interaction of electromagnetic waves with polar solvent molecules, metal salts, and organic linkers in solution resulting in the production of heat, which can then also be transferred conductively—the kinetics of nucleation and crystallisation are also likely to vary.^26^, ^27^ In many instances, the use of microwave heating leads to the reduction of particle size, resulting in nanoparticles with a narrow size distribution,^28^, ^29^, ^30^ suggested to be a consequence of more homogeneous heating of the reaction solution leading to faster nucleation kinetics,^31^, ^32^ and effectively inducing LaMer burst nucleation.^33^, ^34^ As rapid nucleation of crystalline MOF particles occurs, reactant is quickly removed from the synthesis, limiting the reactant available for the growth of particles. However, there are several examples of microwave heating resulting in particles of the same size to those produced solvothermally.^22^ For example, when UiO‐67, the isoreticular analogue of UiO‐66 with biphenyl‐4,4’‐dicarboxylate linkers, is produced through an l‐proline modulated synthesis, the particle size shows no change with varying heat source, suggesting modulators retain a considerable effect on the nucleation and crystal growth process when undergoing microwave heating.^12^, ^35^

To determine crystallisation behaviour under solvothermal conditions, turbidity measurements offer a simple alternative to in situ diffraction techniques,^36^, ^37^, ^38^ which often require bespoke equipment and synchrotron access. The homogeneous starting solution allows the user to pinpoint crystallisation as light transmissivity decreases upon the crystallisation of solid material. This methodology has been effectively used to monitor the nucleation of covalent organic framework (COF) synthesis,^39^ and very recently, alongside spectroscopic techniques, the mechanism of formation of MIL‐53(Al).^40^ Herein, we probe the crystallisation process of UiO‐66 by turbidity measurements in a parallel crystallisation system. Multiple reaction parameters are probed, from temperature and concentration to the addition of modulators, and their effects on the kinetics of crystallisation collated. Following these results, the insight gained from turbidity measurements is used to analyse the rapid microwave synthesis of UiO‐66, once more varying reaction parameters to better understand the crystallisation process.

## Results and Discussion

### Crystallisation kinetics

Experimental runs proceeded through the reported UiO‐66 solvothermal synthesis,^16^ consisting of a 1:1 ratio of ZrCl_4_ and BDC in *N*,*N*‐dimethylformamide (DMF, unpurified ACS grade unless otherwise stated) at a range of concentrations, divided into sixteen sealed 1 mL vials. To mimic typical laboratory syntheses of UiO‐66, standard laboratory grade reagents and solvents were used without purification. These vials are then simultaneously heated within the Crystal16 parallel crystalliser and held at the desired reaction temperature, with turbidity measurements monitoring the crystallisation process. Each run yields 16 independent measurements of the induction time, defined as the time until detection of crystals under constant process conditions like temperature, from which the induction time probability distribution can be determined, producing a plot of induction time probability versus time. The generated solids were collected, combined, and examined by powder‐X‐ray diffraction to confirm the formation of UiO‐66 (see Supporting Information, Section S2). In addition, previous studies investigating synthesis and modulation of UiO‐66 have shown no transient species crystallising prior to UiO‐66.^7^, ^19^, ^20^ It should be noted that some run‐to‐run variability in absolute induction time was observed; careful attempts were made to limit experimental variability within a single run as much as possible (inter alia)—the data represented in each individual figure were collected at the same time using the same reagents—and the trends observed are consistent, allowing valid comparisons to be made. We are therefore confident that the stochastic nature of nucleation^37^ is the primary reason for variations in specific induction time distributions.

**Figure 2:**
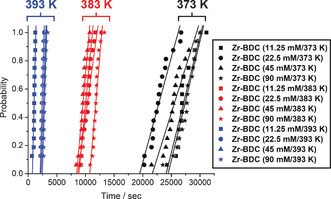
Dependence of UiO‐66 nucleation time on temperature and concentration in ACS grade DMF.

The first parameter studied was that of reaction temperature. MOF syntheses in formamide solvents are believed to be driven by the thermal decomposition of the reaction solvent. As the solvent (in this case DMF) breaks down,^41^ a base (in this case dimethylamine) is released, which is of sufficient basicity to deprotonate the organic linker, allowing for assembly with the inorganic cluster ultimately forming the framework. As a result, increasing the reaction temperature should lead to a faster nucleation rate, as has been observed for DMF‐based syntheses of Zr‐fum by EDXRD.^20^ Solutions of varying concentration were thus run at 373 K, 383 K and 393 K to probe the effect of temperature and to validate the turbidity measurement protocol, and the samples are named **Zr‐BDC (concentration/temperature)** to denote the conditions probed. The measurements produce induction time distributions exemplified by those in Figure 2, where it is possible to assess the onset time—the appearance of the first data point—and the variation in the overall distribution of the points. We hypothesise that a larger time of onset is caused by a slower rate of reaction and a larger variation is due to a slower nucleation rate.

As expected, the onset of the induction time distribution occurs more rapidly as the reaction temperature is increased, with materials synthesised at 373 K showing an average induction time of about 25 000 s compared to approximately 2300 s for the 393 K syntheses, as a consequence of the more rapid decomposition of DMF at higher temperatures. Reduction in temperature also appears to introduce greater variance in induction times, with material synthesised at 373 K crystallising over a larger time range, suggestive of slower nucleation, compared to at 393 K.

Whilst maintaining a 1:1 ratio of Zr^4+^ to ligand, syntheses with concentrations ranging from 11.25 mm to 90 mm were carried out across the temperature range. A general trend was observed, whereby higher concentration solutions typically displayed longer induction times. Although this result appears initially counterintuitive, this could be due to the higher concentration of ZrCl_4_ in the synthesis leading to a greater release of HCl, potentially hindering the deprotonation of the carboxylate linker and leading to slower assembly. Interestingly, the increase in concentration did not reduce significantly the variance in the induction time distribution, indicating that nucleation rates were similar.

As synthesis temperature was shown to greatly affect the rate of crystal nucleation, further reaction parameters were then examined at a constant temperature of 393 K. For subsequent experiments, the higher concentration syntheses (90 mm) were replaced with an intermediate concentration (33.75 mm) due to solubility issues when certain additives were used, as homogeneous starting solutions are essential for accurate nucleation determination. All experiments were carried out at 393 K, and so temperature is not denoted in sample names forthwith. The addition of water is routinely used in the synthesis of zirconium based MOFs, with the nucleation of tetragonal ZrO_2_ nanoparticles implied as a precursor to forming the hexanuclear secondary building unit.^42^ A lack of water can also lead to the formation of an alternative Zr terephthalate MOF, MIL‐140A,^43^ at higher temperatures (MIL‐140A is said to be the thermodynamic product, while UiO‐66 is the kinetic one, in this system). Certainly, a source of oxygen for the bridging oxo and hydroxo units in the clusters of both Zr terephthalates is required, and often not controlled for: sources of adventitious water may include partially hydrolysed or hydrated metal salts, wet solvents, or absorption of atmospheric moisture.

**Figure 3:**
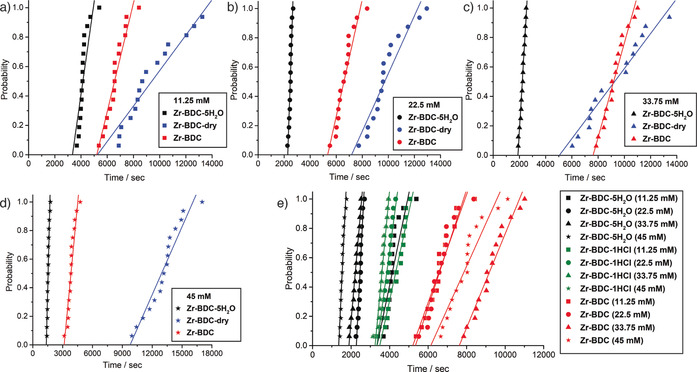
The effect of water on the nucleation time of UiO‐66 at 393 K, probed by using dry DMF, dry DMF spiked with water, and reagent grade DMF at reaction concentrations of a) 11.25 mm, b) 22.5 mm, c) 33.75 mm, and d) 45 mm. An aggregated plot of all data is given in the Supporting Information (Figure S1). e) The effect of addition of HCl on the nucleation time of UiO‐66 across all concentrations compared to the previous data. Individual plots for each concentration are given in the Supporting Information (Figure S2).

The use of dry DMF (water content <0.005 %, **Zr‐BDC‐dry**, blue symbols in Figure 3) led to overall longer induction times compared with reagent grade DMF (water content <0.2 %, **Zr‐BDC**, red symbols in Figure 3) over the reaction concentration range studied, and higher concentration solutions again generally nucleated more slowly. Whilst it is difficult to ensure completely dry conditions, these results show a reduced presence of water hinders the nucleation and formation of UiO‐66. Syntheses using dry DMF deliberately spiked with 5 equiv of water (**Zr‐BDC‐5H_2_O**, black symbols in Figure 3) show a vast decrease in the induction time, which occurs after only 1300 s for **Zr‐BDC‐5H_2_O (45 mM)**, and with all 16 vials routinely nucleating within a very short distribution time frame compared with the **Zr‐BDC‐dry** experiments. In contrast to our previous observations, the induction onset time now shows a general increase with decreasing reagent concentration, complying with conventional nucleation theories that predict higher nucleation rates at increased reagent concentrations. This could indicate that the rate of formation of the metal clusters, aided by the addition of water, overrides the effect of the increased release of HCl by the starting materials (and indeed concomitant consumption of water) at higher concentrations, which limits deprotonation of the linker.

The effect of HCl on crystallisation was examined (Figure 3 e) via the addition of 1 equiv of 37 % HCl to the reaction solutions using the same dry DMF solvent (**Zr‐BDC‐1HCl**, green symbols in Figure 3 e). Addition of HCl results in quicker onset of nucleation than the control, **Zr‐BDC**, with an induction time of approximately 3600 s for **Zr‐BDC‐1HCl (11.25 mM)** and higher concentrations tending towards quicker crystallisation. As previously outlined, the addition of an external HCl source is commonly thought to speed up the crystallisation process,^21^ which these results appear to confirm, as the addition of 37 % HCl results in concomitant addition of about 3.3 moles of water per mole of HCl. However, nucleation onset occurs later than for the **Zr‐BDC‐5H_2_O** samples, suggesting that HCl can also slow nucleation by modifying reaction pH and hindering linker deprotonation. These results are a further indication that the rate of crystallisation may depend more heavily on the rate of formation of the metal clusters rather than the diprotonation of the linker.

**Figure 4:**
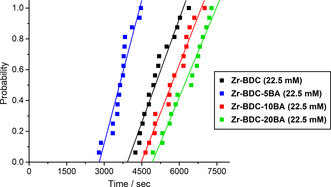
Effect of the addition of benzoic acid on the induction time of UiO‐66.

Monocarboxylic acid modulators, such as benzoic and acetic acid, have become routine components in the syntheses of zirconium MOFs, helping to achieve highly crystalline material while tailoring properties such as particle size and defectivity.^12^, ^13^ The addition of modulators of this kind is theorised to slow crystallisation by coordinative competition and reversibility, leading to the formation of larger sized particles, although it may also induce Zr_6_ cluster formation and could influence reaction pH. The effect of benzoic acid concentration on the induction time was determined; the addition of 5 equiv of benzoic acid (Figure 4) led to an unexpected faster onset of nucleation, with **Zr‐BDC‐5BA (22.5 mM)** crystallising approximately 1800 s before **Zr‐BDC (22.5 mM)**, suggesting a complex interplay of different processes. At this lower concentration, the modulator may be aiding in pre‐organising the Zr_6_ SBUs in the pre‐crystallisation solutions, but not be at a high enough concentration to inhibit the onset of nucleation. As the benzoic acid concentration is increased to 10 and 20 equiv, the induction time can be seen to increase, with the onset being latest for **Zr‐BDC‐20BA (22.5 mM)**, suggesting at higher modulator concentrations, the dominant effect is coordinative competition of the modulator for the Zr sites.

It became apparent throughout the course of the study that repeating experiments on different days with different solutions often resulted in discrepancies between absolute induction times, although the reported trends are highly reproducible. It has previously been observed that aging stock solutions of precursors affects physical properties such as particle size and porosity of the resultant UiO‐66 material formed.^44^ The time taken between preparing solutions and running turbidity measurements at increased temperature could be a key experimental variable, but aging of the reagents before solution preparation was also thought to be a potential notable variable. For example, the possibility of ZrCl_4_ hydrolysing in humid air, reacting with one mole of H_2_O to form ZrOCl_2_ and two moles of HCl, is a process which may lead to reproducibility issues as potential molecular influences on kinetics are consumed (H_2_O) and released (HCl).^45^ Additionally, ZrOCl_2_ is thought to be a precursor to the hexanuclear SBU of UiO‐66; its direct use as source of Zr leads to very small nanoparticles and gels,^20^, ^45^, ^46^ suggesting rapid nucleation.

**Figure 5:**
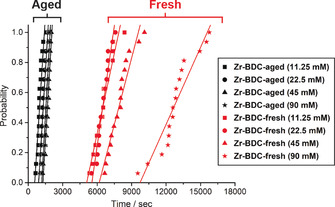
Effect of the aging of the ZrCl_4_ precursor before solution preparation on UiO‐66 induction time distributions.

Repeating turbidity measurements (Figure 5) with a freshly opened bottle of ZrCl_4_ (**Zr‐BDC‐fresh**, red symbols in Figure 5) caused a significant difference in induction times compared to syntheses using an “aged” bottle (**Zr‐BDC‐aged**, black symbols in Figure 5),^47^ with UiO‐66 nucleating much more slowly in the former case, confirming that partial hydrolysis of aged reagents can dramatically modify nucleation. Attempts to measure induction times by turbidity with ZrOCl_2_ as the Zr source were hampered not only by the gelation of the solutions on heating, but also by the production of very small nanoparticles reducing the accuracy of the turbidity measurement. Qualitatively, induction times decreased substantially—the highest solution concentration crystallised before the hold temperature of 393 K could even be reached—confirming the effect of changing Zr source to ZrOCl_2_.

Despite the large differences in overall crystallisation time between **Zr‐BDC‐aged** and **Zr‐BDC‐fresh** experiments, the trend of the higher concentration solutions crystallising slower is still apparent, along with low temperature leading to slower crystallisation. The differences in absolute induction time across the experiments demonstrate the complexity of the crystallisation process of UiO‐66, with slight differences in reagents potentially resulting in vast differences in nucleation. However, the induction time distributions reproduce the trends and influences observed previously by in situ diffraction work, confirming that the technique is valuable for examining MOF nucleation behaviour. A summary of the different influences on nucleation kinetics is given in Table 1.

**Table 1:** Summary of the studied variables, their effect on UiO‐66 self‐assembly, and rationalisation of each effect.

Variable	Effect on Nucleation		
	Onset Time	Variability	Rationale
temperature	earlier onset	less variability	more rapid DMF decomposition and release of base to deprotonate linker
increased reagent concentration	later onset	no change in variability	greater presence of HCl from higher ZrCl_4_ concentration lowers pH and hinders linker deprotonation
water content	earlier onset, with concomitant reversal of concentration effect	less variability	more rapid hydrolysis of ZrCl_4_ and greater provision of O^2−^ and OH^−^ ligands for SBU formation
HCl addition (pH)	earlier onset due to water content; less significant than water alone	variability decreases as reagent concentration increases	lowers pH but also introduces water to synthesis; competing effects, but water addition has greater influence
modulation	earlier onset at low modulator concentration, later at higher	no change in variability	multiple competing processes in solution; SBU pre‐organisation would give earlier onset, but competition with linkers would give later onset
aging of ZrCl_4_	earlier onset	less variability	partial hydrolysis of ZrCl_4_ during aging effectively adds water to synthesis

### Microwave synthesis

Following the study into how synthesis parameters alter the rate at which nucleation occurs, conditions were transferred to microwave assisted rapid synthesis. Microwave heating is common in MOF synthesis, producing highly crystalline material in a fraction of the time.^22^, ^23^ Whilst this heating mode does differ to conventional heating, we aimed to implement knowledge gained from the induction time study in order to facilitate rapid synthesis. It is postulated that the difference in heating, for example more localised, direct heating within the microwave compared with conventional heating, is the leading reason for reduced synthesis times. It should be noted that the induction times account for a 20 min temperature ramp period, with time 0 being the point at which the synthesis temperature is achieved, rather than the onset of heating. Comparatively, a sample in a conventionally heated oven will have a much longer initial heating period due to the increased size of both the oven and sample volume. Microwave prepared samples, however, have a much reduced heating period, reaching the required temperature in as little as 2 min. This difference in heating rate should be kept in mind when comparing synthesis methods.

**Figure 6:**
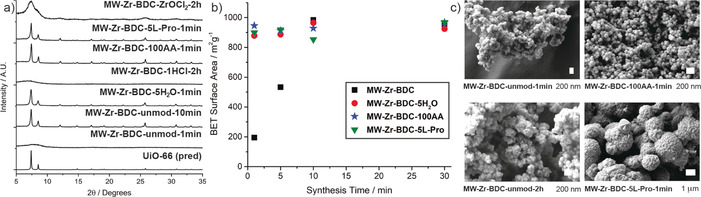
a) Stacked PXRD patterns of selected microwave syntheses compared with the pattern for UiO‐66 predicted from CSD deposition RUBTAK.^17^ b) Plot of BET surface area (N_2_, 77 K) versus reaction time, showing that modulation in all cases produces crystalline UiO‐66 more rapidly at short reaction time. c) SEM images of selected samples showing the differing particle size and morphology induced by modulation.

The syntheses of **Zr‐BDC (45 mM)**, **Zr‐BDC‐5H_2_O (45 mM)** and **Zr‐BDC‐1HCl (45 mM)** were repeated in the microwave at 393 K (see Supporting Information, Section S3), increasing the scale from 1 mL to 10 mL, to give **MW‐Zr‐BDC‐Unmod**, **MW‐Zr‐BDC‐5H_2_O**, and **MW‐Zr‐BDC‐1HCl**, in turn. Samples modulated with 100 equivalents of acetic acid (**MW‐Zr‐BDC‐100AA**) were also prepared to assess the effect of modulation. In order to ensure a close comparison, reaction parameters were kept as close as possible when changing from Crystal16 to the microwave; 45 mm concentration solutions of ZrCl_4_ and BDC in a 1:1 ratio, with reaction times ranging from 1 min to 2 h. The solids were assessed by PXRD, N_2_ adsorption, and scanning electron microscopy (SEM), and notable differences were observed in the different microwave syntheses, varying with both reaction time and synthetic conditions (see summary in Figure 6 and comprehensive data in the Supporting Information).

The **Zr‐BDC (45 mM)** conditions were successfully reproduced via microwave heating (**MW‐Zr‐BDC‐Unmod**), producing crystalline material in as little as 5 min, although the crystallinity of the 5 min sample is lessened compared to the samples of longer synthesis times. When synthesis time was reduced to 1 min the resultant material was amorphous, as shown by PXRD (Figure 6 a, and Supporting Information Figure S4). The surface areas of the materials were determined by BET analysis, with samples produced in 10 min to 2 h ranging from 889 m^2^ g^−1^ to 984 m^2^ g^−1^, about 80 % of the calculated surface area of pristine UiO‐66.^23^ As the synthesis time is reduced to 5 min, the surface area drops to 533 m^2^ g^−1^, with a further drop to 195 m^2^ g^−1^ when synthesis time is reduced to 1 min (Figure 6 b). This large decrease in surface area can be attributed to the production of an increasing amount of non‐porous amorphous material as the synthesis time decreases.

**MW‐Zr‐BDC‐Unmod‐2 hr** shows an approximate particle size of 200 nm, with generally well‐defined octahedral morphology, however, a considerable amount of the sample is intergrown, forming agglomerates (Figure 6 c). As the synthesis time decreases, the definition of the material can also be seen to decrease, with **MW‐Zr‐BDC‐Unmod‐10 min** being considerably more intergrown, and **MW‐Zr‐BDC‐Unmod‐1 min** lacking the distinctive octahedral shape, instead producing agglomerates of spherical material, as expected from the amorphous PXRD pattern.

Modulated syntheses typically resulted in more rapid microwave synthesis of crystalline UiO‐66, as can be seen by plotting the BET surface areas of the samples versus synthesis time, which is consistent with the turbidity data (Figure 6 b). Doping syntheses with 5 equiv of water (**MW‐Zr‐BDC‐5H_2_O**), led to the successful synthesis of material in as little as 1 min. A reduction in particle size to approximately 100 nm is also observed, implying fast nucleation followed by limited growth as reactants are used up in the nucleation step. The surface areas of **MW‐Zr‐BDC‐5H_2_O** samples are again slightly lower than predicted, ranging from 791 m^2^ g^−1^ at 2 hrs to 965 m^2^ g^−1^ at 10 min. The thermal stability of both the unmodulated and water doped samples was examined, with all materials showing very high thermal stability up to 773 K, typical of UiO materials (see Supporting Information, Figure S5).^48^

Surprisingly, the addition of 1 equiv HCl in the microwave synthesis did not produce significant amounts of crystalline material regardless of synthesis time (**MW‐Zr‐BDC‐1HCl)**, with PXRD patterns lacking distinct peaks and suggesting amorphous samples, although small reflections consistent with UiO‐66 may be visible, likely corresponding to a small amount of nanocrystalline UiO‐66 amongst primarily amorphous material (see Supporting Information, Figure S6 and exemplar in Figure 6 a). The IR spectra of **MW‐Zr‐BDC‐1HCl** samples also lack distinctive bands, while SEM images show agglomerates of poorly defined particles of approximately 300 nm. N_2_ adsorption analysis of **MW‐Zr‐BDC‐1HCl‐1 min** and **MW‐Zr‐BDC‐1HCl‐2 hr** suggest that the samples are not be completely void of space, with surface areas of 374 m^2^ g^−1^ and 360 m^2^ g^−1^, respectively, further suggestive of a mixture of some crystalline UiO‐66 and some amorphous coordination polymer material. The same conditions subjected to 24 h heating in a conventional oven produces crystalline material; transferring conventional solvothermal syntheses to microwave heating is clearly not a guarantee of success.

**Figure 7:**
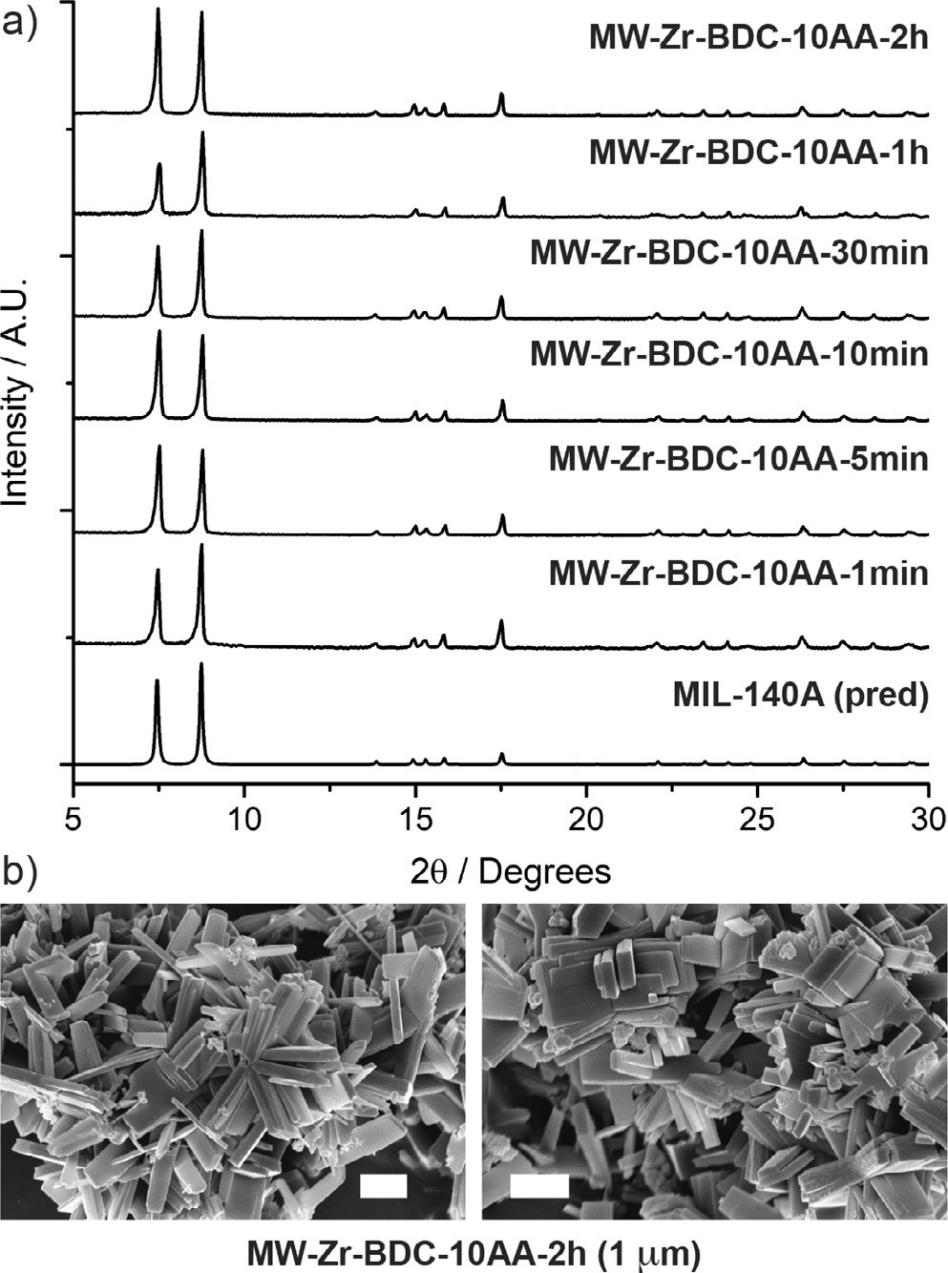
a) Stacked PXRD patterns for **MW‐Zr‐BDC‐10AA** compared with that predicted for MIL‐140A (CSD deposition ZONBAH),^43^ confirming the formation of MIL‐140A rather than UiO‐66. b) SEM images showing tetragonal plate morphology of selected samples, characteristic of MIL‐140A. Scale bars 1 μm.

The addition of 100 equiv acetic acid, also routinely used in Zr MOF syntheses, enabled the synthesis of highly crystalline material (**MW‐Zr‐BDC‐100AA**) in as quickly as 1 min (Figure 6 a). The surface areas of **MW‐Zr‐BDC‐100AA** show good agreement with the previous samples, ranging from 946 m^2^ g^−1^ at 1 min to 1087 m^2^ g^−1^ at 2 h, with the isotherms suggesting considerable interparticle space (Figure 6 b, and Supporting Information, Figure S7). SEM shows there to be a good particle size distribution, with particles being approx. 100 nm and becoming more discrete rather than intergrown agglomerates (Figure 6 c). Upon attempts to repeat the synthesis through conventional heating, crystallisation was not achieved within the 24 h synthesis time, likely as the acetic acid content is relatively high compared with other previously reported modulated syntheses of UiO‐66.^13^, ^14^, ^49^ As such, synthesis with 10 equiv of acetic acid was attempted through both conventional and microwave heating; **Zr‐BDC‐10AA** under conventional heating produced UiO‐66, while **MW‐Zr‐BDC‐10AA** reproducibly yielded MIL‐140A, [ZrO(BDC)]_*n*_, a more condensed phase with infinite one‐dimensional ZrO SBUs^43^ (Figure 7). Typical syntheses for MIL‐140A require much higher temperatures, reaching up to 220 °C, with suggestion that MIL‐140 is the thermodynamic product and UiO‐66 the kinetic.^43^, ^50^ In this case, the temperature is the lowest reported so far for isolation of MIL‐140A.

Our analogous work with Fe MOFs has shown that modulation can induce formation of the thermodynamic over the kinetic product,^51^ which may explain the low temperature synthesis of MIL‐140A in this case. Microwave heating has also previously been proposed to selectively produce *kinetic* phases rather than thermodynamic phases; synthesis of MIL‐53(Cr) and MIL‐101(Cr), both formed from chromium precursors and terephthalic acid, produces mixed phases via conventional heating, while microwave heating selectively produces phase‐pure MIL‐101(Cr), the kinetically favoured product, most likely due to the faster kinetics of nucleation and crystallisation.^52^

Clearly modulation complicates this process further, as microwave heating in this case forms the *thermodynamic* product preferentially. This is further illustrated by the formation of UiO‐66 at lower (**MW‐Zr‐BDC‐Unmod**) and higher acetic acid concentrations (**MW‐Zr‐BDC‐100AA**), suggesting some modulator‐induced pre‐clustering in the latter, and this is under further investigation.

Following our previous use of l‐proline in solvothermal reactions, as well as the microwave synthesis of UiO‐67, we assessed the convertibility to the microwave synthesis of UiO‐66 (**MW‐Zr‐BDC‐5L‐Pro**).^12^ With 5 equiv of l‐proline added, alongside 1 equiv of HCl as used in our previous report, we were again able to produce crystalline material in as little as 1 minute, with particle size ranging from 50 nm to 500 nm, showing large spherical agglomerates of particles with characteristic octahedral morphology (Figure 6 c, and Supporting Information, Figure S8). Thermogravimetric analysis shows a gradual mass loss starting from 473 K, indicating the inclusion of proline/proline derivatives within the framework. This is confirmed by ^1^H NMR spectroscopy of digested samples, as peaks for proline and formyl‐proline can be seen, along with formate peaks (Supporting Information, Figure S9). Despite this, the surface areas of **MW‐Zr‐BDC‐5L‐Pro** samples do not drop compared with previous samples, suggesting l‐proline to be incorporated into the framework rather than the pores, along with potentially an increase in defects. Similar samples produced solvothermally have recently been utilised as organocatalysts.^53^

As expected, the use of zirconyl chloride (**MW‐Zr‐BDC‐ZrOCl_2_**) results in particularly small nanoparticles of <20 nm in diameter, which is reflected in the broad PXRD patterns, and indicative of rapid nucleation (Supporting Information, Figure S10). Scherrer analysis of the main Bragg reflection at 2*θ*=7.32° gives a particle size of 10.4 nm, correlating well with the SEM images. Nanocrystal formation is consistent with the use of zirconyl chloride in solvothermal syntheses which also produces particularly small particles.^20^, ^46^ These conditions led to the synthesis of a gel, which upon washing and drying gave a solid pellet which was ground down for analysis. Characterisation of the material produced in reduced synthesis times matches closely with that of previously reported solvothermal material, confirming the convertibility of these reaction parameters.

## Conclusions

The crystallisation kinetics of UiO‐66 have been investigated using turbidity measurements, a comparatively simple and fast technique which has been validated as an alternative route to probe MOF self‐assembly compared to in situ diffraction analysis. Insight was gained into how several reaction parameters, such as temperature and the use of common modulators, affect the speed of crystallisation. Varying reaction temperature had a large effect on the rate of crystallisation, with a reduction from 393 K to 373 K leading to considerably slower formation of material. The use of both water and HCl as modulators sped up crystallisation, further confirming the same conclusions drawn by Ragon et al. in their energy‐dispersive X‐ray diffraction study.^20^ Carboxylate‐containing modulators showed more complex behaviour, whilst the age of reagents, notably the hydrolysis of ZrCl_4_, also resulted in significant variability in nucleation time. We expect that similar aging of DMF—breakdown, water absorption, and so on—will also induce variability, and so should be controlled in future studies.

The information gained from the turbidity experiments on the effect of modulator addition on nucleation onset was used to aid the rapid synthesis of UiO‐66 through microwave assisted heating. Various syntheses were examined, each taking reaction time down to one minute, with addition of modulators resulting in more rapid synthesis of crystalline, porous UiO‐66. Despite reports of microwave synthesis often leading to smaller particles due to rapid nucleation, UiO‐66 did not appear to show a reduction in particle size with reduced synthesis time. However, surface areas were slightly lower than expected, suggesting rapid synthesis may result in some poorly crystalline or amorphous by‐products. Two notable differences were observed comparing conventional heating to microwave synthesis. Firstly, HCl did not effectively modulate microwave synthesis of UiO‐66 in our hands, producing mostly amorphous material. Secondly, a rapid, low temperature, microwave‐induced, acetic acid modulated synthesis of the alternative phase MIL‐140A was discovered, suggesting that combining modulation with microwave heating could lead to enhanced efficient syntheses of new phases and control between kinetic and thermodynamic products in the self‐assembly of MOFs.

## Conflict of interest

The authors declare no conflict of interest.

## Supporting information

As a service to our authors and readers, this journal provides supporting information supplied by the authors. Such materials are peer reviewed and may be re‐organized for online delivery, but are not copy‐edited or typeset. Technical support issues arising from supporting information (other than missing files) should be addressed to the authors.

Supplementary
