# Supplementary material for: Assessing Crystallisation Kinetics of Zr Metal–Organic Frameworks through Turbidity Measurements to Inform Rapid Microwave‐Assisted Synthesis
Source: Chemistry. 2020 May 11;26(30):6910–8. doi: 10.1002/chem.202000993 (PMC7318326; doi:10.1002/chem.202000993)
Supplement: Supplementary file 1 — Supplementary [file CHEM-26-6910-s001.pdf]

Chemistry–A European Journal

Supporting Information

Assessing Crystallisation Kinetics of Zr Metal–Organic Frameworks through Turbidity Measurements to Inform Rapid Microwave-Assisted Synthesis

Sarah L. Griffin,[a, b] Maria L. Briuglia,[b] Joop H. ter Horst,*[b] and Ross S. Forgan*[a]

Assessing Crystallisation Kinetics of Zr Metal-Organic Frameworks through Turbidity Measurements to Inform Rapid Microwave-Assisted Synthesis

Sarah L. Griffin,[a,b] Maria L. Briuglia,[b] Joop ter Hoorst,[b,*] Ross S. Forgan[a,*]

a. WestCHEM School of Chemistry, University of Glasgow, Glasgow, UK.
Email: ross.forgan@glasgow.ac.uk

b. EPSRC Centre for Innovative Manufacturing in Continuous Manufacturing and Crystallisation, University of Strathclyde Technology and Innovation Centre, Glasgow, UK.
Email: joop.terhorst@strath.ac.uk

SUPPORTING INFORMATION

Table of Contents:

S1. General Methods and Materials **S2.**
S2. Crystal16 Experimental Procedure **S4.**
S3. Microwave Synthesis **S10.**
S4. References **S21.**

S1. General Methods and Materials

All reagents and chemicals were purchased from Sigma-Aldrich, Fischer Scientific, Tokyo Chemical Industry, Acros Organics, Fluorochem and Alfa Aesar and used without further purification. Solvents were purchased from Sigma-Aldrich, VWR Internationals, Acros Organics and Fischer Scientific.

Powder X-ray Diffraction (PXRD): PXRD measurements were collected at 298 K using a PANalytical X'Pert powder diffractometer (λ (CuKα) = 1.4505 Å). Data was collected over the range 3 – 45 ° on a mounted bracket sample stage. (University of Glasgow)

Thermal Gravimetric Analysis (TGA): Measurements were carried out using a TA Instruments Q500 Thermogravimetric Analyser. Measurements were collected from room temperature to 800 °C with a heating rate of 10 °C/min under an air atmosphere. (University of Glasgow)

Nuclear Magnetic Resonance (NMR): NMR spectra were recorded on either a Bruker AVIII 400 MHz spectrometer or a Bruker AVI 500 MHz spectrometer, and referenced to residual solvent peaks. (University of Glasgow)

Gas Uptake: N_2 adsorption isotherms were carried out at 77 K on a Quantachrome Autosorb iQ gas sorption analyser. Prior to analysis, samples were degassed at 120 °C for 20 hours using the internal turbo pump. BET surface areas were calculated from the isotherms using the Micropore BET Assistant implemented in the Quantachrome ASiQwin operating software. (University of Glasgow)

Scanning Electron Microscope (SEM): Powder samples were deposited onto conductive carbon tabs and coated with Pd for 50 seconds using Polaron SC7640 sputter coater. The prepared samples were transferred to and imaged with a Carl Zeiss Sigma Variable Pressure Analytical SEM with Oxford Microanalysis. (University of Glasgow)

Infrared Spectroscopy (IR): Infrared spectra of solids were obtained via a Shimadzu Fourier Transform Spectrometer, FTIR-8400S, fitted with a Diamond ATR unit. (University of Glasgow)

Crystal16: Crystal16 multi-reactor system, with a transmissivity source wavelength of 645 nm and heating rate of 5 °C/min. (Centre for Continuous Manufacturing and Crystallisation, University of Strathclyde Technology and Innovation Centre)

Microwave: Microwave synthesis was carried out in a CEM Discover SP microwave system, with a power of 200 W.

S2. Crystal16 Experimental Procedure

General Procedure: Separate solutions of zirconium tetrachloride and terephthalic acid were prepared. The corresponding quantities were added to 25 mL jars along with reagent grade DMF (10 mL), followed by sonication to ensure full dissolution and a homogeneous solution. Immediately prior to reaction, 0.5 mL of each solution was aliquoted into sixteen 1 mL HPLC vials equipped with stirrer bars. The sealed vials were placed in the Crystal16 reactor and heated simultaneously at a rate of 5 °C/min to the set temperature. The moment the set temperature was reached was taken as time zero. The moment the transmission of light through the sample decreased from 100% was taken as the induction time for that sample. Following synthesis, material from the sixteen individual vials was collected and combined, washing with DMF (x2) and acetone (x2) before drying. Basic reagent quantities are listed in Table S1.

Table S1. Reagent quantities for Crystal16 experiments run at differing concentrations.

Conc (mM)	$ZrCl_4$ (g)	BDC (g)
11.25	0.0525	0.0374
22.50	0.1049	0.0748
33.75	0.1576	0.1122
45.00	0.2098	0.1496
90.00	0.4196	0.1496

S2.1 Influence of Temperature

Runs were carried out at 373 K, 383 K, and 393 K for concentrations 11.25 mM, 22.5 mM, 45 mM, and 90 mM using reagent grade DMF. Reactions are named **Zr-BDC (concentration / temperature)**, and the data are plotted in Figure 2 of the main manuscript.

S2.2 Influence of Water and HCl

All subsequent reactions were carried out at 393 K, so no temperature is noted in reaction names. **Zr-BDC** uses reagent grade DMF, **Zr-BDC-dry** uses dry DMF (<0.005% water), **Zr-BDC-5H_2O** uses the same dry DMF and is spiked with five equivalents of distilled water, while **Zr-BDC-1HCl** uses dry DMF spiked with 1 equivalent of concentrated (37%) HCl (Table S2).

Table S2. Reagent and modulator quantities for Crystal16 experiments run at differing concentrations.

Conc (mM)	$ZrCl_4$ (g)	BDC (g)	5H_2O (μL)	1HCl (μL)
11.25	0.0525	0.0374	20	20
22.50	0.1049	0.0748	40	40
33.75	0.1576	0.1122	60	60
45.00	0.2098	0.1496	80	80

Full experimental procedures follow.

Zr-BDC (11.25 – 45 mM). Separate solutions of zirconium tetrachloride and terephthalic acid were prepared. The corresponding quantities were added to 25 mL jars along with reagent grade DMF (10 mL), followed by sonication to ensure full dissolution and a homogeneous solution. Immediately prior to reaction, 0.5 mL of each solution was aliquoted into sixteen 1 mL HPLC vials equipped with stirrer bars. The sealed vials were placed in the Crystal16 reactor and heated simultaneously at a rate of 5 °C/min.

Zr-BDC-5H_2O (11.25 – 45 mM). Separate solutions of zirconium tetrachloride and terephthalic acid were prepared. The corresponding quantities were added to 25 mL jars along with dry DMF (10 mL), followed by sonication to ensure full dissolution and a homogeneous solution. Immediately prior to reaction, the appropriate volume of water was added to the jar containing the zirconium chloride solution, and briefly sonicated. 0.5 mL of each solution was aliquoted into sixteen 1 mL HPLC vials equipped with stirrer bars. The sealed vials were placed in the Crystal16 reactor and heated simultaneously at a rate of 5 °C/min.

Zr-BDC-dry (11.25 – 45 mM). Separate solutions of zirconium tetrachloride and terephthalic acid were prepared. The corresponding quantities were added to 25 mL jars along with dry (<0.002% water) DMF (10 mL), followed by sonication to ensure full dissolution and a homogeneous solution. Immediately prior to reaction, 0.5 mL of each solution was aliquoted into sixteen 1 mL HPLC vials equipped with stirrer bars. The sealed vials were placed in the Crystal16 reactor and heated simultaneously at a rate of 5 °C/min.

The data for these samples are plotted for each individual concentration in Figure 3 in the main manuscript. A complete comparison of all data is shown in Figure S1.

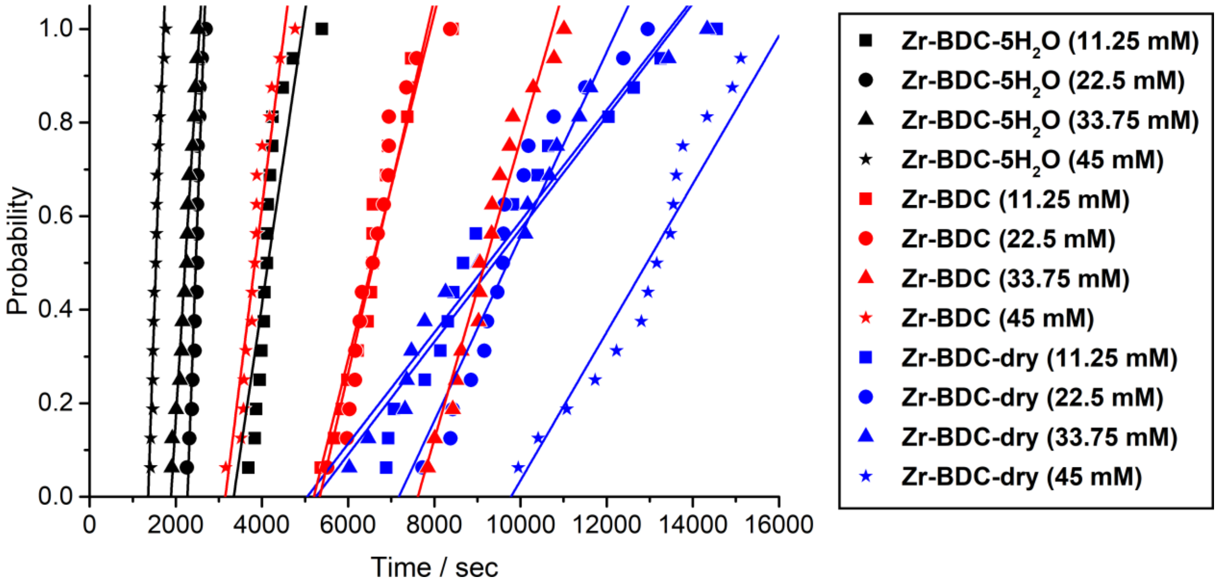

Figure S1. Probability of crystallisation at 393 K as influenced by the use of dry DMF and the addition of 5 eq. of water to dry DMF.

Zr-BDC-1HCl. Separate solutions of zirconium tetrachloride and terephthalic acid were prepared. The corresponding quantities were added to 25 mL jars along with dry (<0.002% water) DMF (10 mL), followed by sonication to ensure full dissolution and a homogeneous solution. Immediately prior to reaction, the appropriate volume of 37% HCl was added to the jar containing the zirconium chloride solution, and briefly sonicated. 0.5 mL of each solution was aliquoted into sixteen 1 mL HPLC vials equipped with stirrer bars. The sealed vials were placed in the Crystal16 reactor and heated simultaneously at a rate of 5 °C/min.

Data are collated in Figure 3e of the main manuscript. Data for individual concentrations are provided in Figure S2 for comparison.

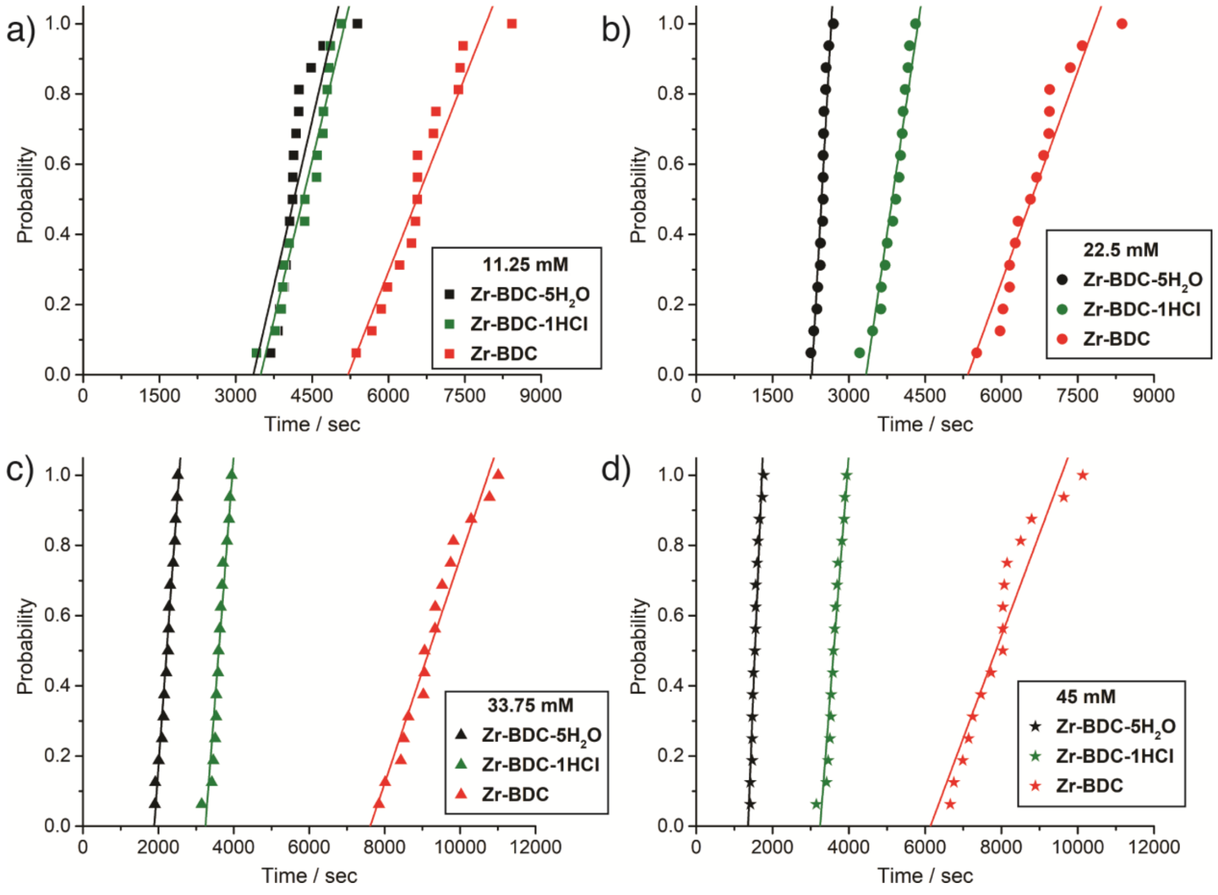

Figure S2. Probability plots comparing the effects of water and HCl on crystallisation time, with syntheses carried out at 393 K.

S2.3. Influence of Modulator

Zr-BDC-BA (5 – 20 Equivs). Separate solutions of zirconium tetrachloride and terephthalic acid were prepared. The corresponding quantities were added to 25 mL jars along with reagent grade DMF (10 mL), followed by sonication to ensure full dissolution and a homogeneous solution at a concentration of 22.5 mM (Table S3). Immediately prior to reaction, the appropriate quantity of benzoic acid was added to the jar containing the zirconium chloride solution, and sonicated to ensure homogeneity. 0.5 mL of each solution was aliquoted into sixteen 1 mL HPLC vials

equipped with stirrer bars. The sealed vials were placed in the Crystal16 reactor and heated simultaneously at a rate of 5 °C/min.

Table S3. Reagent and Benzoic Acid quantities for Crystal16 experiments run at 22.5 mM.

Conc (mM)	$ZrCl_4$ (g)	BDC (g)	5BA (g)	10BA (g)	20BA (g)
22.50	0.1049	0.0748	0.2748	0.5495	1.0991

S2.4. Influence of the Age of $ZrCl_4$

The “aged” zirconium chloride used in this study was estimated to have been in use for over a year, whereas the “fresh” experimental run used newly opened zirconium tetrachloride which was subsequently kept in a dry box.

Zr-BDC-aged/fresh. Separate solutions of zirconium tetrachloride and terephthalic acid were prepared. The corresponding quantities were added to 25 mL jars along with reagent grade DMF (10 mL), followed by sonication to ensure full dissolution and a homogeneous solution. 0.5 mL of each solution was aliquoted into sixteen 1 mL HPLC vials equipped with stirrer bars. The sealed vials were placed in the Crystal16 reactor and heated simultaneously at a rate of 5 °C/min. The values used are the same as those listed in Table S1.

S2.5. PXRD Characterisation

Samples from individual Crystal16 runs were combined and isolated by centrifugation after washing with DMF. Powder X-ray Diffraction analysis confirmed formation of UiO-66 in all cases; representative data for selected examples are shown in Figure S3.

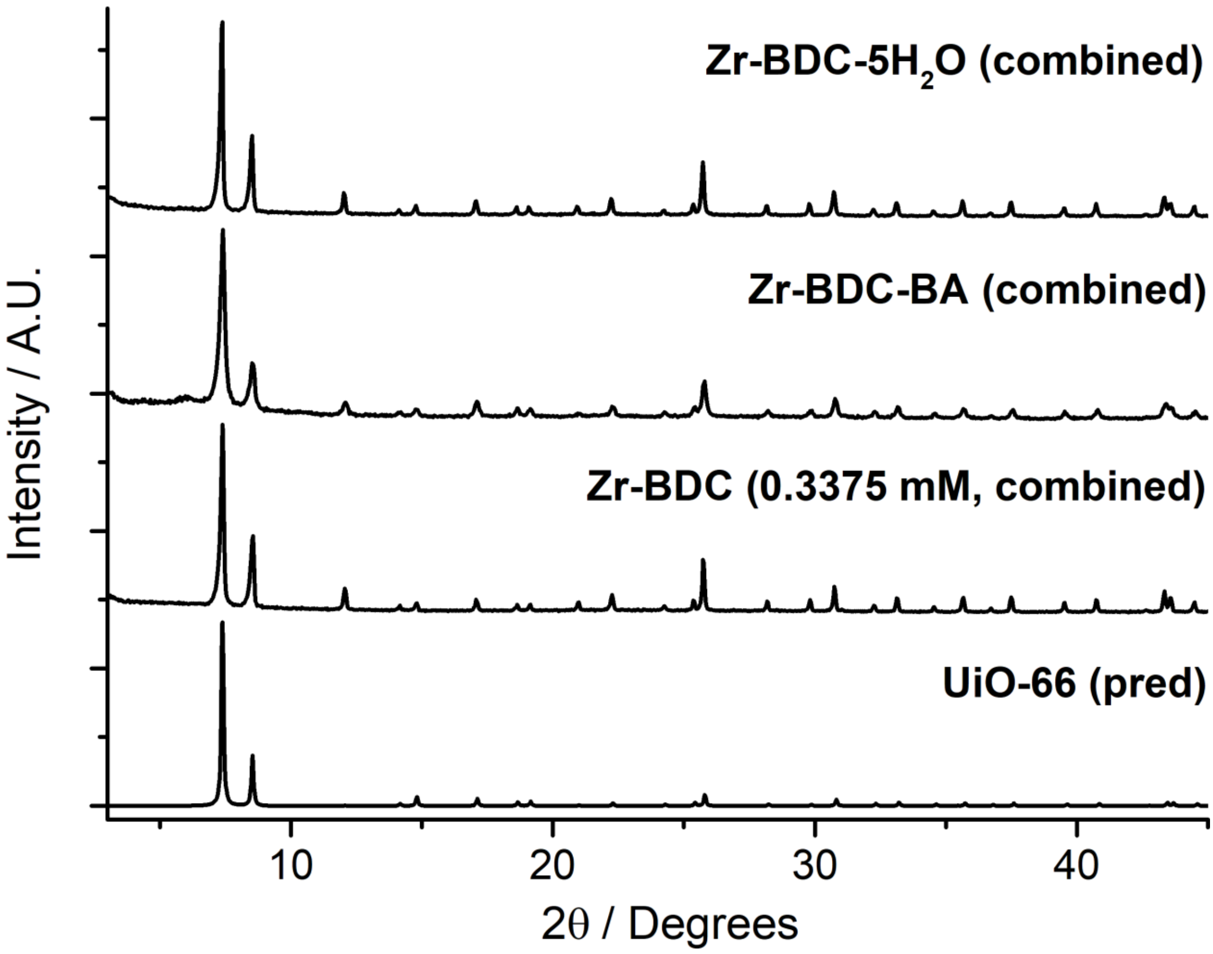

Figure S3. Stacked powder patterns of combined material produced from various Crystal16 experiments as compared to the predicted pattern for UiO-66 (CSD deposition RUBTAK),[S1] confirming the production of highly crystalline UiO-66.

S3. Microwave Synthesis

Microwave syntheses of Zr-BDC MOFs were carried out under a range of modulation conditions, detailed in the sample naming scheme in Table S4.

Table S4. Naming scheme for microwave syntheses.

**Microwave synthesis conditions**	**Abbreviated name**
Unmodulated	**MW-Zr-BDC-Unmod**
Water 5 eq	**MW-Zr-BDC-5H_2O**
HCl 1 eq	**MW-Zr-BDC-1HCl**
Acetic acid 100 eq	**MW-Zr-BDC-100AA**
Acetic acid 10 eq	**MW-Zr-BDC-10AA**
Proline + HCl	**MW-Zr-BDC-5L-Pro**
Zirconyl chloride	**MW-Zr-BDC-$ZrOCl_2$**

MW-Zr-BDC-Unmod. Terephthalic acid (0.45 mmol, 0.0748 g) and zirconium tetrachloride (0.45 mmol, 0.1049 g) were added to a 35 ml microwave vial followed by 10 ml reagent grade DMF. The solution was sonicated until dissolved, stirred for 5 mins then heated at 100 °C for the allotted time. Upon completion the material was washed with DMF (x2) and acetone (x2) before drying. To ensure full activation the material was soaked in chloroform for three days, exchanging solvent each day, followed by heating at 120 °C for 20 hr under high vac. Characterisation of the samples is given in Figure S4.

MW-Zr-BDC-5H_2O. Terephthalic acid (0.45 mmol, 0.0748 g) and zirconium tetrachloride (0.45 mmol, 0.1049 g) were added to a 35 ml microwave vial followed by 10 ml reagent grade DMF. The solution was sonicated until all material was fully dissolved, followed by the addition of water (40 µL). The solution was stirred for 5 mins then heated at 100 °C for the allotted time. Upon completion the material was washed with DMF (x2) and acetone (x2) before drying. To ensure full activation the material was soaked in chloroform for three days, exchanging solvent each day, followed by heating at 120 °C for 20 hr under high vac. Characterisation of the samples is given in Figure S5.

MW-Zr-BDC-1HCl. Terephthalic acid (0.45 mmol, 0.0748 g) and zirconium tetrachloride (0.45 mmol, 0.1049 g) were added to a 35 ml microwave vial followed by 10 ml reagent grade DMF. The solution was sonicated until all material was fully dissolved, followed by the addition of 37% HCl (40 μL). The solution was stirred for 5 mins then heated at 100 °C for the allotted time. Upon completion the material was washed with DMF (x2) and acetone (x2) before drying. To ensure full activation the material was soaked in chloroform for three days, exchanging solvent each day, followed by heating at 120 °C for 20 hr under high vac. Characterisation of the samples is given in Figure S6.

MW-Zr-BDC-100AA. Terephthalic acid (0.45 mmol, 0.0748 g) and zirconium tetrachloride (0.45 mmol, 0.1049 g) were added to a 35 ml microwave vial followed by 10 ml reagent grade DMF. The solution was sonicated until all material was fully dissolved, followed by the addition of acetic acid (2.5 mL). The solution was stirred for 5 mins then heated at 100 °C for the allotted time. Upon completion the material was washed with DMF (x2) and acetone (x2) before drying. To ensure full activation the material was soaked in chloroform for three days, exchanging solvent each day, followed by heating at 120 °C for 20 hr under high vac. Characterisation of the samples is given in Figure S7.

MW-Zr-BDC-10AA. Terephthalic acid (0.45 mmol, 0.0748 g) and zirconium tetrachloride (0.45 mmol, 0.1049 g) were added to a 35 ml microwave vial followed by 10 ml reagent grade DMF. The solution was sonicated until all material was fully dissolved, followed by the addition of acetic acid (260 μL). The solution was stirred for 5 mins then heated at 100 °C for the allotted time. Upon completion the material was washed with DMF (x2) and acetone (x2) before drying. To ensure full activation the material was soaked in chloroform for three days, exchanging solvent each day, followed by heating at 120 °C for 20 hr under high vac. Characterisation of the samples, confirming they are MIL-140A, is given in Figure 7 of the main text.

MW-Zr-BDC-5L-Pro. Terephthalic acid (0.45 mmol, 0.0748 g), zirconium tetrachloride (0.45 mmol, 0.1049 g) and L-proline (2.25 mmol, 0.259 g) were added to a 35 ml microwave vial followed by 10 ml reagent grade DMF. The solution was sonicated until all material was fully dissolved, followed by the addition of HCl (40

μL). The solution was stirred for 5 mins then heated to 100 °C for the allotted time. Upon completion the material was washed with DMF (x2) and acetone (x2) before drying. To ensure full activation the material was soaked in chloroform for three days, exchanging solvent each day, followed by heating at 120 °C for 20 hr under high vac. Characterisation of the samples is given in Figure S8. To assess modulator incorporation, samples were digested in DMSO-d_6 and D_2SO_4 under heating, and examined by ^{1}H NMR Spectroscopy, shown in Figure S9.

MW-Zr-BDC-ZrOCl$_2$. Terephthalic acid (0.45 mmol, 0.0748 g) and zirconyl chloride octahydrate (0.45 mmol, 0.145 g) were added to a 35 ml microwave vial followed by 10 ml DMF. The solution was sonicated until dissolved, stirred for 5 mins then heated at 100 °C for the allotted time. Upon completion the gel material was washed with DMF (x2) and acetone (x2) before drying. The resultant pellet was ground to a powder. To ensure full activation the material was soaked in chloroform for three days, exchanging solvent each day, followed by heating at 120 °C for 20 hr under high vac. Characterisation of the samples is given in Figure S10.

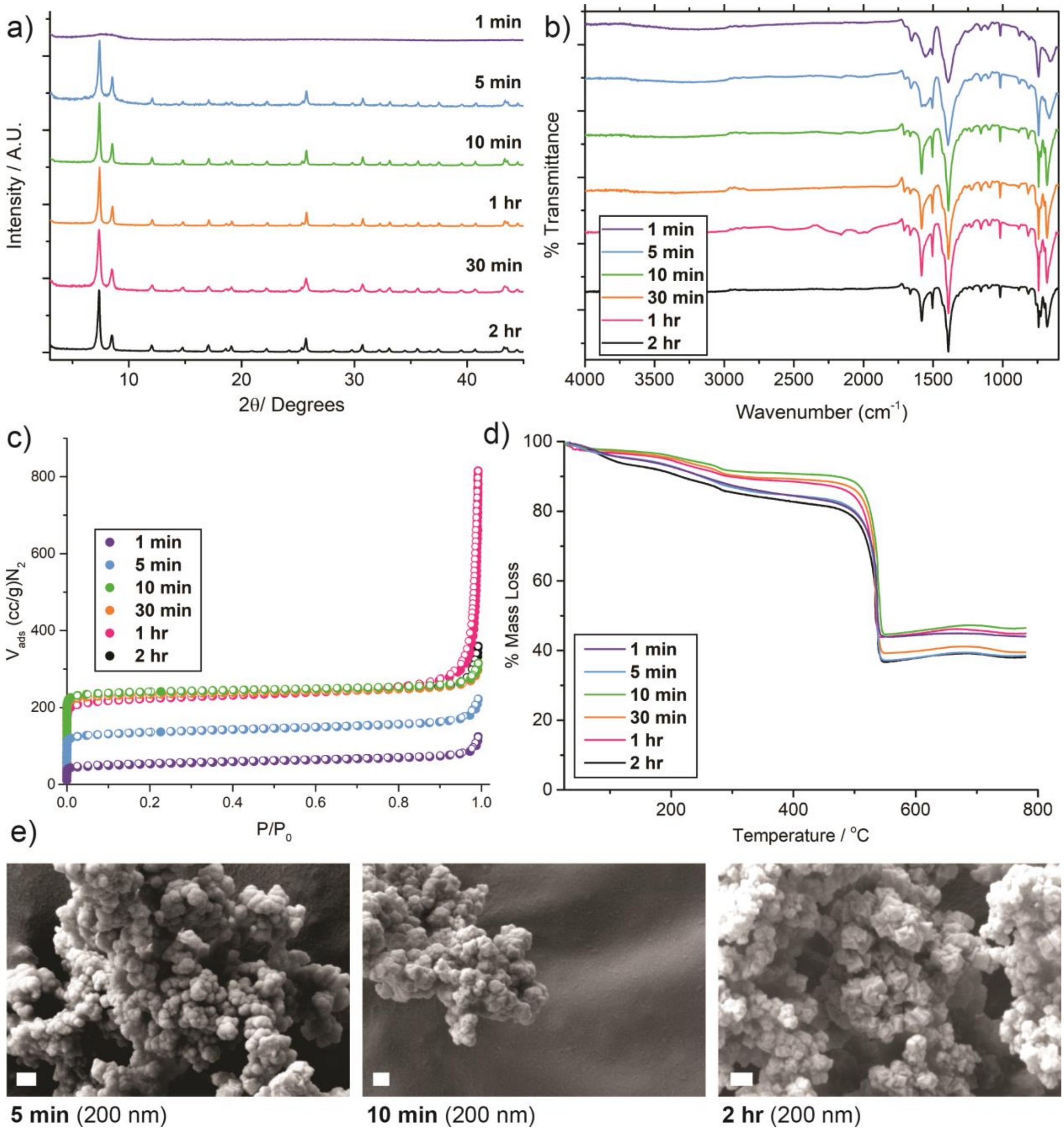

Figure S4. Characterisation of **MW-Zr-BDC-Unmod**. a) Stacked powder patterns confirming crystallinity of material produced in 5 min - 2 hr syntheses, with the 1 min sample being amorphous. b) IR spectra of the resulting materials, showing broad, less distinct spectra for sample of poorer crystallinity. c) N_2 uptake isotherms (77 K, filled symbols adsorption, empty symbols desorption) showing increased surface area with increased reaction time (BET surface areas for all samples are collated in Table S5). d) TGA showing the thermal stability of the materials, decomposing above 500 °C. e) SEM images of selected samples, showing greater definition for longer reaction times.

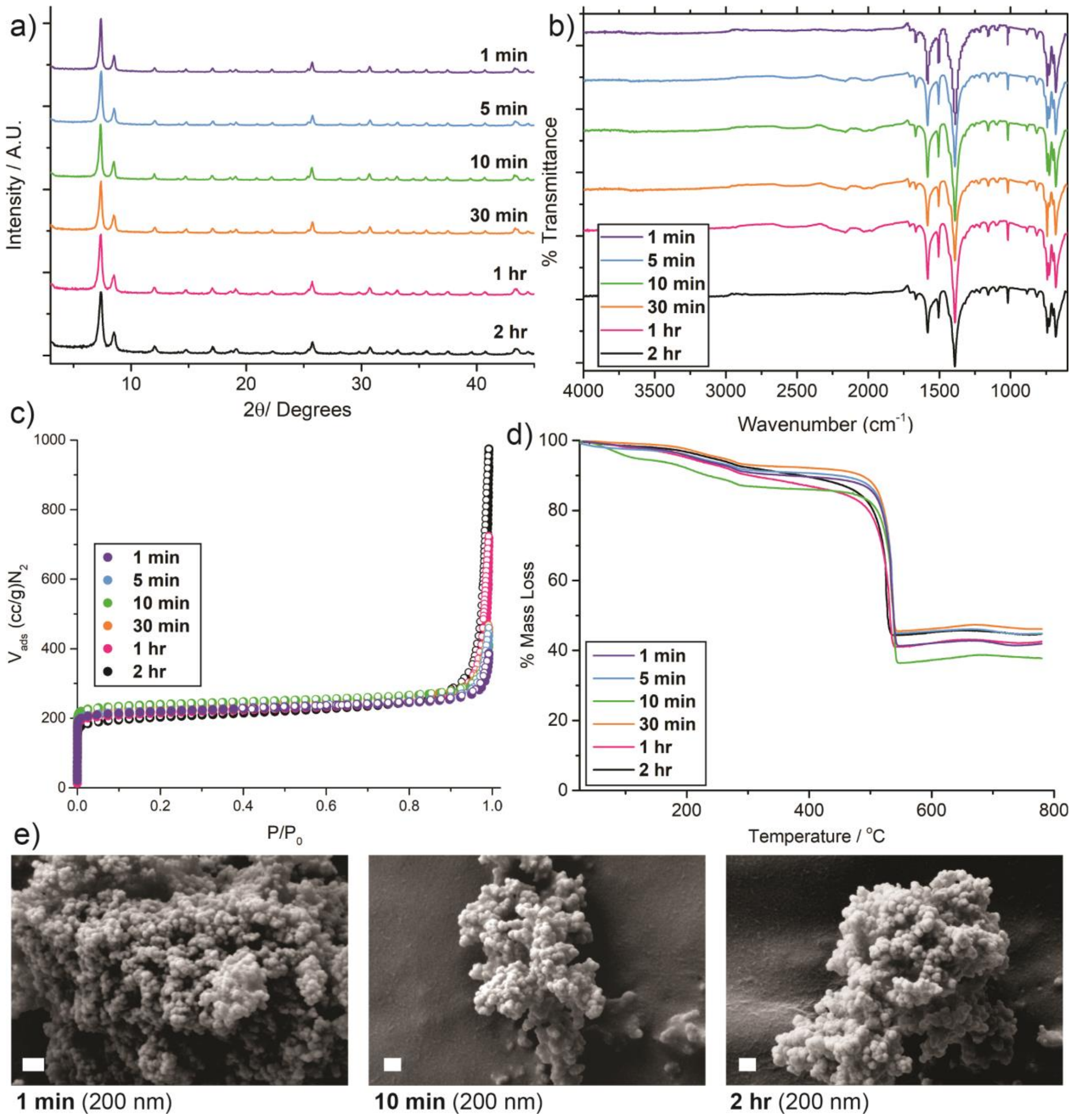

Figure S5. Characterisation of **MW-Zr-BDC-5H_2O**. a) Stacked powder patterns confirming crystallinity of all materials produced in 1 min - 2 hr. b) IR spectra of the resulting materials. c) N_2 uptake isotherms (77 K, filled symbols adsorption, empty symbols desorption) showing no notable changes in surface area with increased reaction time (BET surface areas for all samples are collated in Table S5). d) TGA showing the thermal stability of the materials, decomposing above 500 °C. e) SEM images of selected samples, showing small ill-defined nanoparticles across all reaction times.

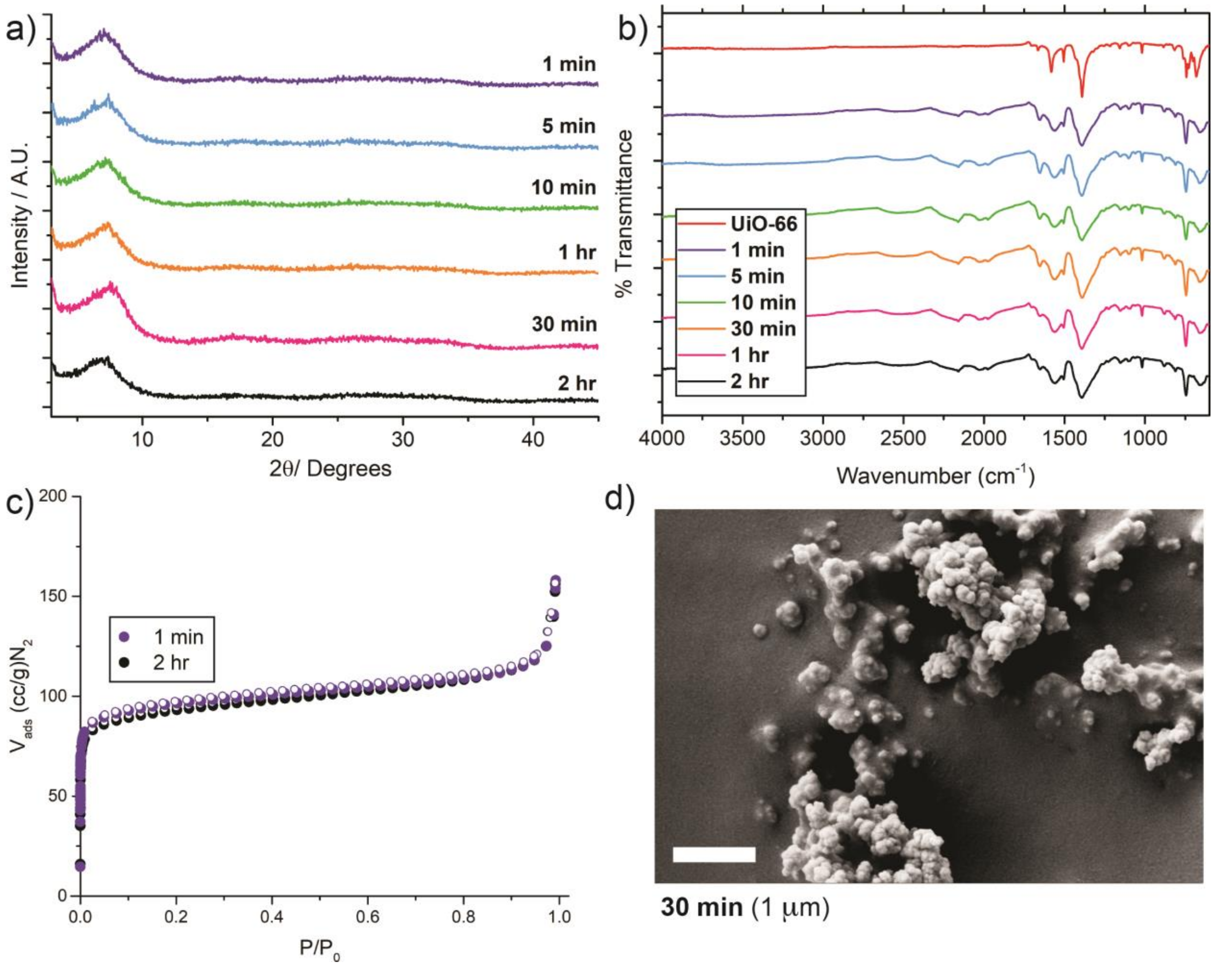

Figure S6. Characterisation of **MW-Zr-BDC-1HCl**. a) Stacked powder patterns showing all samples to be almost entirely amorphous. b) IR spectra showing broad and poorly defined bands which suggest similar metal-ligand connectivity to UiO-66 but an amorphous structure. c) N_2 adsorption showing the limited porosity of the 1 min and 2 hr samples, with BET surface areas of approximately 350 m^2g^{-1}, suggesting a small component of the sample may be crystalline UiO-66 (BET surface areas for all samples are collated in Table S5). d) SEM image of **MW-Zr-BDC-1HCl-30min**, showing poorly defined agglomerates which are characteristic across the series.

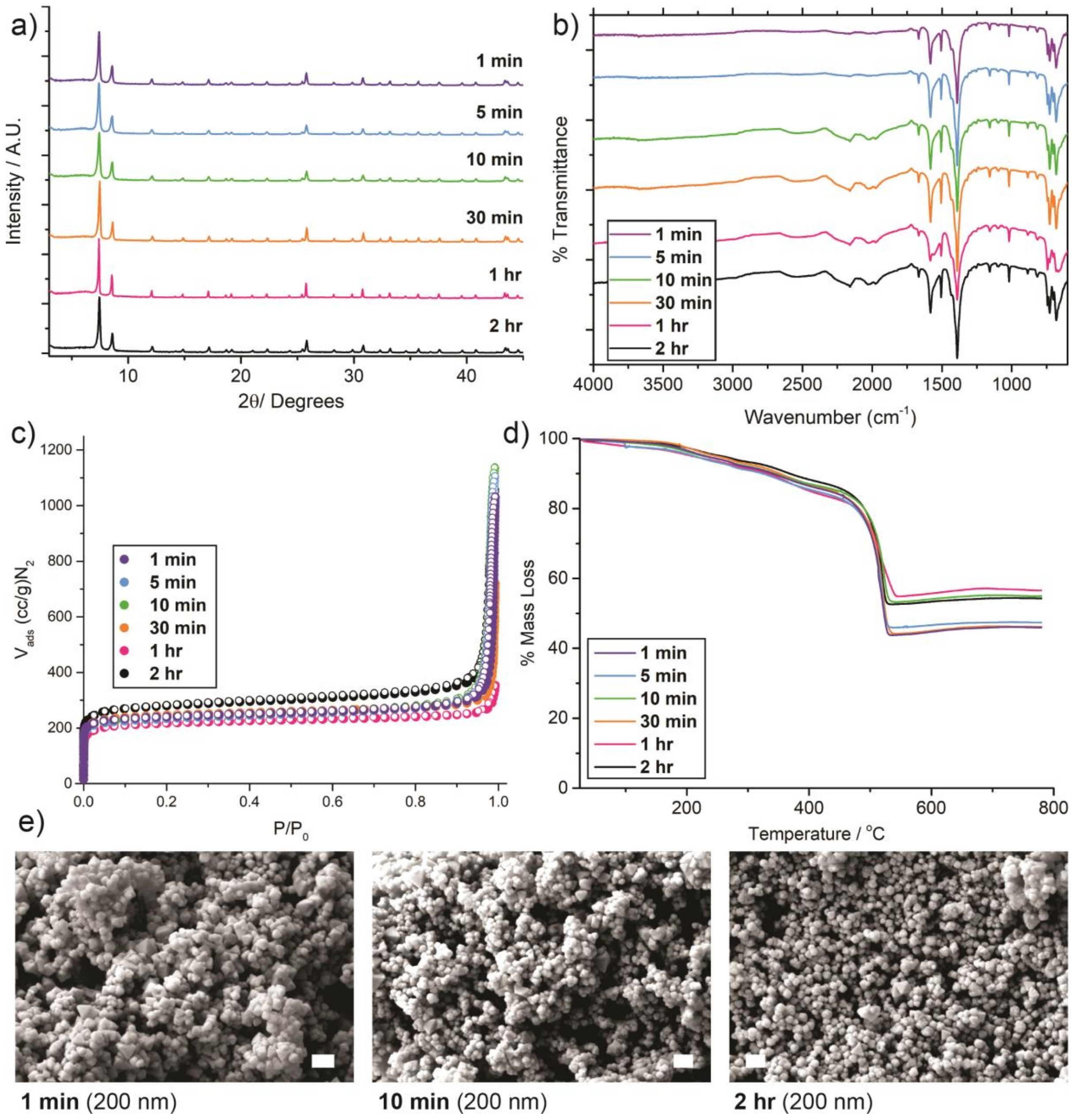

Figure S7. Characterisation of **MW-Zr-BDC-100AA**. a) Stacked powder patterns confirming crystallinity of all materials produced in 1 min - 2 hr. b) IR spectra of the resulting materials. c) N_2 uptake isotherms (77 K, filled symbols adsorption, empty symbols desorption) showing no notable trends in surface area change with increased reaction time (BET surface areas for all samples are collated in Table S5). d) TGA showing the thermal stability of the materials, decomposing above 500 °C. e) SEM images of selected samples, showing small octahedral nanoparticles of similar size across all reaction times.

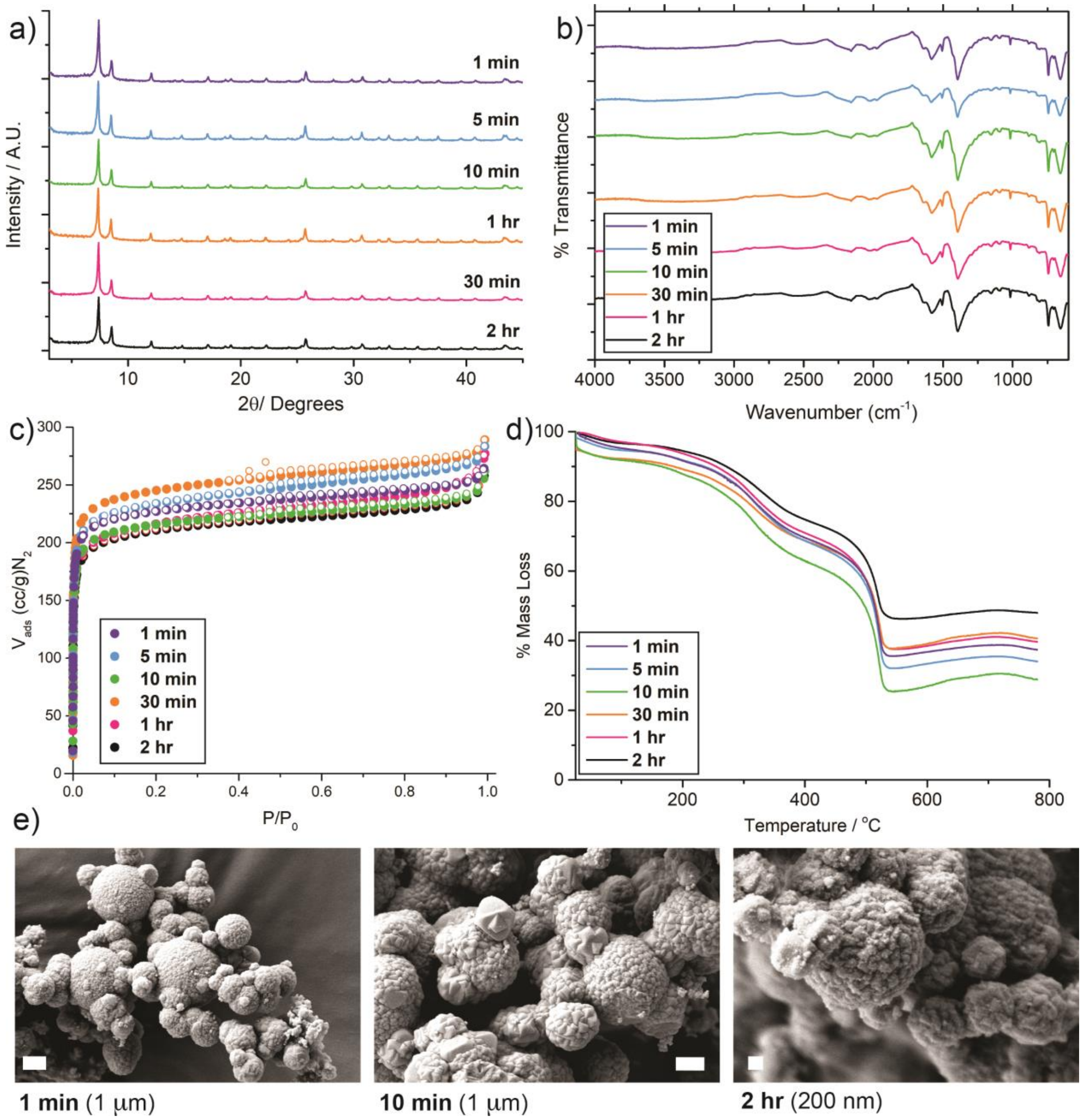

Figure S8. Characterisation of **MW-Zr-BDC-5L-Pro**. a) Stacked powder patterns confirming crystallinity of all materials produced in 1 min - 2 hr. b) IR spectra of the resulting materials. c) N_2 uptake isotherms (77 K, filled symbols adsorption, empty symbols desorption) showing no notable trends in surface area change with increased reaction time (BET surface areas for all samples are collated in Table S5). d) TGA showing the thermal stability of the materials, decomposing above 500 °C, but with additional mass loss around 300-400 °C suggesting incorporation of L-proline at defect sites. e) SEM images of selected samples, showing spherical agglomerates of small octahedral particles across all reaction times.

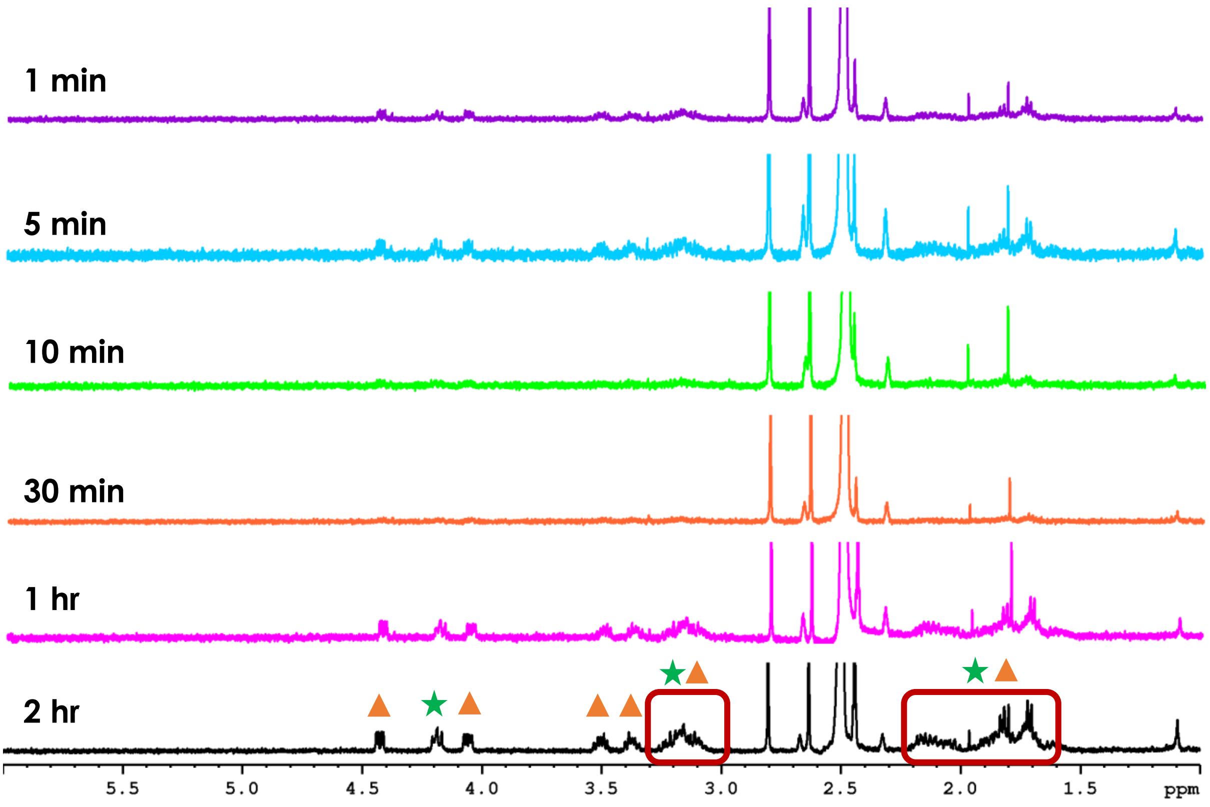

Figure S9. Stacked ^{1}H NMR spectra of digested **MW-Zr-BDC-5L-Pro** samples, with signals resulting from incorporated proline (star) and formyl proline (triangle) highlighted. The formylation of L-Proline is consistent with literature observations.[S2] Signals within red boxes correspond to both proline and formyl proline, but cannot be distinguished from one another due to overlap. Spectra are calibrated to residual DMSO solvent peak. There appears to be no trend with regards to levels of proline incorporation in the samples over different times.

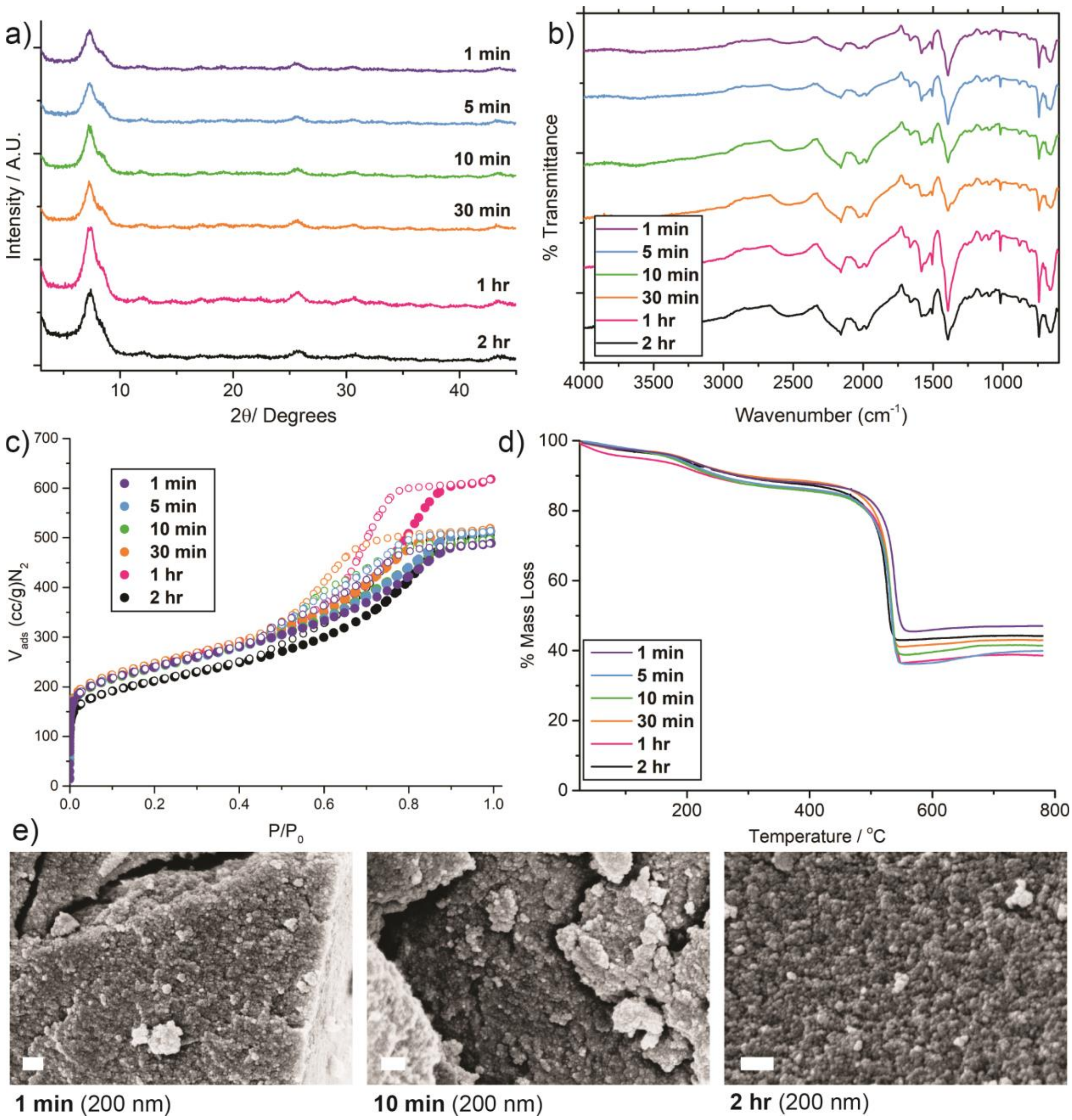

Figure S10. Characterisation of **MW-Zr-BDC-$ZrOCl_2$**. a) Stacked powder patterns with broad Bragg peaks suggesting formation of UiO-66 with very low particle size across all experiments. b) IR spectra of the resulting materials. c) N_2 uptake isotherms (77 K, filled symbols adsorption, empty symbols desorption) showing no notable trends in surface area change with increased reaction time, but with significant mesoporosity associated with interparticle spacing between very small nanoparticles (BET surface areas for all samples are collated in Table S5). d) TGA showing the thermal stability of the materials, decomposing above 500 °C. e) SEM images of selected samples, showing nanoparticles of less than 20 nm forming monolithic structures across all reaction times.

Table S5. Collated BET surface areas (N_2, 77 K, $m^2 g^{-1}$) for all microwave samples.

Time	Unmod	$5H_2O$	1HCl	100AA	5L-Pro	$ZrOCl_2$
**1 min**	195	878	374	946	899	862
**5 min**	533	887	—	913	919	864
**10 min**	984	965	—	928	853	857
**30 min**	955	924	—	968	968	889
**1 hr**	889	857	—	848	844	878
**2 hr**	961	791	360	1047	826	762

S4. References

[S1] J. H. Cavka, S. Jakobsen, U. Olsbye, N. Guillou, C. Lamberti, S. Bordiga and K. P. Lillerud, *J. Am. Chem. Soc.* **2008**, *130*, 13850-13851.

[S2] X. Feng, H. S. Jena, K. Leus, G. Wang, J. Ouwehand and P. Van Der Voort, *J. Catal.* **2018**, *365*, 36-42.
